# Potential limitations of diagnostic standard codes to distinguish polycythemia vera and secondary erythrocytosis

**DOI:** 10.1038/s41598-022-08606-1

**Published:** 2022-03-18

**Authors:** Alanna Barrios-Ruiz, Daniel Davila-Gonzalez, Eric Fountain, Lee Cheng, Srdan Verstovsek, Cristhiam M. Rojas-Hernandez

**Affiliations:** 1 Instituto Tecnológico Y de Estudios Superiores de Monterrey, Monterrey, Mexico; 2 Division of Cancer Medicine, The University of Texas MD Anderson Cancer Center, Houston, TX USA; 3 Institute of Cancer Care Innovation, The University of Texas MD Anderson Cancer Center, Houston, TX USA; 4 Department of Leukemia, Division of Cancer Medicine, The University of Texas MD Anderson Cancer Center, Houston, TX USA; 5 Section of Benign Hematology, The University of Texas MD Anderson Cancer Center, 1515 Holcombe Blvd. Suite 1464, Houston, TX 77030 USA

**Keywords:** Haematological cancer, Medical research

## Abstract

Red cell overproduction is seen in polycythemia vera (PV), a bone marrow myeloproliferative neoplasm characterized by trilinear cell proliferation (WBC, platelets), as well as in secondary erythrocytosis (SE), a group of heterogeneous disorders characterized by elevated EPO gene transcription. We aimed to verify the concordance of the International Classification of Diseases (ICD) code-based diagnosis of “polycythemia” or “erythrocytosis” with the true clinical diagnosis of these conditions. We retrospectively reviewed the electronic medical records (January 1, 2005, to December 31, 2016) of adult patients with ICD codes of polycythemia and/or erythrocytosis who had testing done for the presence of the JAK2V617F mutation. We verified the accuracy of the ICD code-based diagnoses by meticulous chart review and established whether these patients fulfilled the criteria by the evaluating physician for PV or SE and according to the World Health Organization 2016 diagnostic guidelines. The reliability of ICD coding was calculated using Cohen's kappa. We identified and chart reviewed a total of 578 patient records. Remarkably, 11% of the patients had concurrent diagnosis codes for PV and SE and were unable to be classified appropriately without individual chart review. The ICD code-based diagnostic system led to misidentification in an important fraction of cases. This represents a problem for the detection of PV or SE cases by ICD-based registries and their derived studies. Research based exclusively on ICD codes could have a potential impact on patient care and public health, and limitations must be weighed when research findings are conveyed.

## Introduction

The term polycythemia denotes an increased total red cell volume, sometimes used interchangeably with erythrocytosis, the former recommended by some experts to be used only in the context of primary myeloproliferative diseases^1^. Erythrocytosis describes an increase above 2 standard deviations in hematocrit or hemoglobin.The measurement of these values is based on whole blood; therefore, it is based on plasma volume and subjects to its changes^2^. To avoid discrepancies, red blood cell mass must be measured for greater accuracy to demonstrate an increase of 125% in sex and body mass, however, this can be difficult to obtain^3^.

Louis Henri Vaquez and Osler were the firsts to make important contributions to primary erythrocytosis, in 2016 WHO redefined the classification and diagnosis criteria of these diseases^4,5^. Primary erythrocytosis, also known as polycythemia vera (PV) is described as the outcome of alterations in the bone marrow that are typically acquired by JAK2 somatic mutations. PV has a characteristic feature of trilineage hematopoietic cell hyperplasia with a congruous bone marrow sample and an increase of hemoglobin level above 16.0 g/dL for females and 16.5 g/dL for males^6,7^. Usually, PV presents with symptoms related to red cell overproduction, pruritus, leukocytosis, thrombocytosis, and splenomegaly. A major call from an international collaboration group recently described the TEMPI syndrome characterized by telangiectasias, erythrocytosis with high EPO expression, monoclonal gammopathy, perinephric fluid collections, and intrapulmonary shunting; due to its clinical presentation it can also be confused with PV^8^.

Secondary erythrocytosis (SE) is a group of heterogeneous disorders, it is mainly caused by abnormal activation of the erythropoietin (EPO) in response to inadequate tissue oxygenation^9^. Therefore, described as an outcome of cardiopulmonary or hypoxia-related issues [smoking, chronic pulmonary disease (COPD), obstructive sleep apnea syndrome (OSA), right to left pulmonary shunts, high altitude habitat], or renal disorders such as arterial stenosis^10^. Acquired causes of this disorder also include hypoxia-independent SE, caused as a side effect of certain diuretic drugs, androgens, anabolic steroids, erythropoietin, post-renal transplantation, or as a result of autonomous EPO production in certain tumors^2^.

The causality of various genetic mutations and their correlation with congenital erythrocytosis cases have been described. Nonsense mutations in the EPO gene receptor, ECYT1 are the lead cause for primary congenital erythrocytosis^3^. The pathogenesis of secondary congenital erythrocytosis involves defects in the oxygen sensing pathway, with multiple target components, notably hypoxia-induced factors, prolyl hydroxylases, and von Hippel-Lindau proteins or EPO gene defects (methemoglobinemia, bisphosphoglycerate mutase deficiency, alteration in α and β globin genes)^2,11^.  The latter mutations are valuable tools to recognize due to important differences in clinical phenotypes, however not always available^11^. Therefore, investigation of pathogenic variants must be made in the presence of early presentation symptoms and positive family history^3^. Molecular advancement could be of importance, to further understanding of idiopathic erythrocytosis (IE) cases.

Erythrocytosis is a common reason for hematology services consultation. The prevalence of PV is estimated to be 47–55 per 100,000 in the United States^12^. This contrasts with the paucity of accurate data on prevalence, mortality, and morbidity of SE, which can be difficult to quantify due to dependent relationships with primary etiologies and insufficient data. Benign hematologists and/or leukemia specialists care for those patients. Despite the expertise and forward understanding of pathophysiology, for some cases, the classification and management of the disease can be far from straightforward and challenging. Moreover, documentation of the appropriate diagnosis is an important task of current medical practice. This implies the use of the international diagnostic classification standard for reporting diseases (ICD codes). ICD codes are widely used in medical and epidemiology research. The introduction of errors at the steps of diagnosis assignment has the potential to introduce systematic mistakes and bias in those types of research studies.

We aimed to describe the concordance of a ten-year ICD code-based cohort of adult patients consulted to our center with a diagnosis of “polycythemia” or “erythrocytosis” (and had testing done for the presence of a JAK2V617F mutation) with the true clinical diagnosis of these conditions, as recorded in the medical records by the evaluating hematologist and supported by the available diagnostic data.

## Methods

This study is a subanalysis of a previously conducted study that aimed to explore the performance of EPO and JAK2 mutations. Our population was selected if they had both tests performed^13^. We reviewed the electronic medical record (EMR) compiled from January 1, 2005, to December 31, 2016 at MD Anderson Cancer Center (MDACC) of patients with ICD-9-CM or ICD-10-CM code diagnosis corresponding to polycythemia (ICD D45) and/or erythrocytosis (ICD D75.1), or similar. We did not include patients with codes for familial erythrocytosis or polycythemia neonatorum.

Patients were selected if the individual medical records (IMR) had at least 2 consecutive billing ICD codes for any of those diagnoses to avoid selecting patients who had an erroneous code entry and those who did not have follow-ups or recurrent visits to complete diagnostic work-up. Additionally, we also excluded patients who did not have JAK2 mutation studies in their medical records.

**Figure 1:**
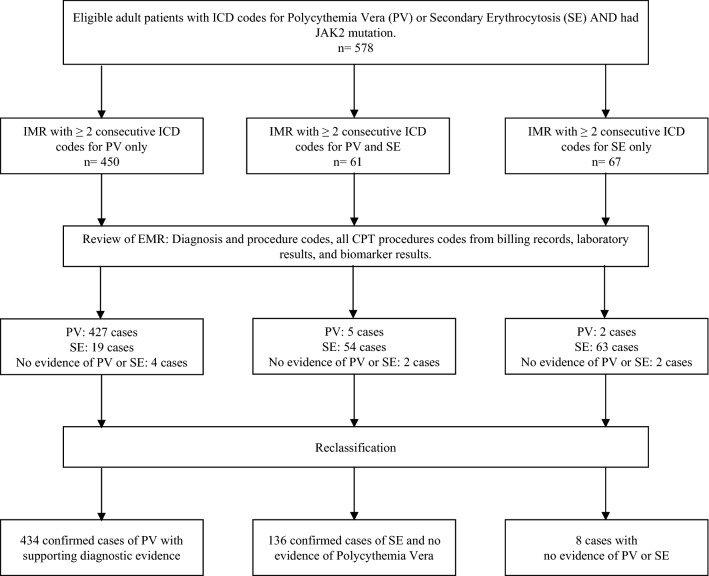
Retrospective review of electronic medical selection and analysis process.

We then reclassified the cases according to 2016 WHO criteria as SE or PV after a meticulous chart review of clinical notes, hematopathology, and other diagnostic data. (Fig. 1). We collected demographic data, comorbidity conditions and medications, relevant laboratory studies including hemoglobin, serum EPO level, JAK 2 mutation status, and bone marrow studies. Records of phlebotomy, polysomnography, tobacco use, and methemoglobin tests were noted.

Data were analyzed using Bioinformatics Core using SAS software and Stata IC statistical software. Variables were described by their frequency, median values, and interquartile range when appropriate. Concordance between ICD and the EMR review reclassification was evaluated by calculating Cohen’s kappa to measure and evaluate the inter-rater reliability agreement according to literature^14^.

The study is a retrospective evaluation of the patients and involved minimal risk to subjects, therefore informed consent was waived by the institutional review board at MD Anderson Cancer Center. The institutional review board at MD Anderson Cancer Center approved the study. All methods were performed in accordance with the relevant guidelines and regulations.

### Ethics approval

The study is a retrospective evaluation of the patients and involved minimal risk to subjects. The institutional review board at MD Anderson Cancer Center approved the study.

## Results

A total of 1092 patients were identified as having at least 2 billing codes for a diagnosis of PV and SE, or both. Of those, 578 corresponded to adult patients (age greater than 18 years of age) who had JAK2 mutation studies. Of those, 480 cases were given an ICD code diagnosis of polycythemia 67 cases with ICD code of erythrocytosis, and 61 cases had both ICD codes used concurrently.

We then chart-reviewed the medical records of the 578 selected cases. Of the patients who had a listed ICD diagnosis of PV, 95% (427) met the WHO criteria for PV; importantly, 4% (19) of those patients had an underlying condition from which the SE diagnosis could have been made (Fig. 1). From the group of cases with only secondary erythrocytosis ICD code, 94% (63) had confirmed SE. Overall, those patients with a single diagnosis code (PV or SE) were “miscoded” in only 5% of cases.

The most notable finding was that 11% of all cases had concurrent codes for both PV and SE. Only through chart review were those cases able to be classified appropriately (Fig. 1). Of those cases, 5 corresponded to PV, 54 to SE, and 2 did not have erythrocytosis.

After medical record review and reclassification of cases, the final population of 434 patients with a confirmed diagnosis of PV had a median age of 59.2 years, 57.72% (329) were male, 86.3% (491) were Caucasian, 3.3% (19) were African American and 10.53% (60) corresponded to other racial groups. A total of 136 patients had a confirmed diagnosis of SE. The median age for that group was 56 years, and 58% of the patients were male (Table 1).

**Table 1:** Demographic and laboratory characteristics of 136 patients with a diagnosis of secondary erythrocytosis at MDACC.

JAK-2 Mutation (V617F PCR) Status	No	%
Positive	2	1.5
Negative	134	98.5

Characteristic	Median	IQR
Age (years)	56.2	66.0–49.0
Male %	57.7	
Female %	42.3	
Serum EPO value (mIU/mL)	19.3	8.8–22.6
Hematocrit %	49.1	24.1﻿–62.0
Hemoglobin (g/dL)	16.7	8.0–20.7

Etiology	Frequency	Percent
Idiopathic erythrocytosis	56	41.18
Hypoxia	44	32.35
Iatrogenic	15	11.03
Autonomous EPO	10	7.35
Multifactorial	6	4.41
Relative polycythemia	5	3.68
Total	136	100

IQR: Interquartile Range.

EPO: Erythropoietin.

Only two patients with SE were noted to have a positive somatic JAK2 V617F mutation by PCR, and they did not have bone marrow morphology criteria compatible with PV. Additionally, 5 patients with SE had other variants (predominantly of germline origin) in the JAK2 gene. JAK2 missense germline variants were reported in 4 cases: 2 (L393 V) gain function, 1 (R1063H), 1 (E846D), and a variant of uncertain origin (R715G). One of those patients had a concurrent germline variant in the TET2 gene.

Concurrent malignancy was found in 56 patients with SE, of which 35.7% had hematological malignancies (non-PV) and 64.2% had solid malignancies. Of the latter, 16.6% had breast cancer, and 13.8% had hepatocellular cancer. The most frequently identified causes of SE in our patients were IE in 56 (41.18%), chronic hypoxic states in 44 (32.4%), iatrogenic erythrocytosis (IaE) in 15 (11%), and autonomous EPO production in 10 (7.35%) (Table 1).

To assess the concordance we calculated the inter-rater reliability (IRR) comparing the ICD diagnosis with the manual review of the dataset through both percent agreement and calculation of Cohen’s Kappa. The Cohen’s Kappa for PV was 0.544 (weak agreement) and for SE was 0.853 (strong agreement), therefore using only ICD diagnosis information to assess databases may lead to the inclusion of SE cases and introduce sample misclassification.

## Discussion

The World Health Organization, International Classification of Diseases, Clinical Modification (ICD-CM) code system, has simplified the extensive amount of information on medical records, improving the identification of diseases, and has an impact on quality-of-care assessment, allocation of resources, evaluation of management patterns and outcomes of diseases. The need for specificity after ICD 9th edition due to the broadness of concepts was overcome with the establishment of the tenth revision in 2014^15^. Despite the above, determining the extent of hematological diseases based solely on hospital discharge codes could be problematic.

Our report highlights the potential limitations of the use of ICD coding for its use in studies. Remarkably, 11% of the patients had a concurrent diagnosis code for PV and SE. Although the percentage of misdiagnosis in this study may appear to be modest, these chronic illnesses have a high cost on health expenses. An analysis made by Metha et al. revealed an age-adjusted prevalence of 56.5 per 100 000 of patients who suffer from polycythemia vera and reported that in 2010, the annual cost reached $14 903 dollars of overall health care, outlining a considerable increase in comorbidities in this population^16^. However, these results, as their authors describe, may have limitations and biases for the same reason as this study. The impact of erroneous adjudication of diagnostic codes may have an impact on interventions using data from public health registries, surveillance and disease control at a population level. It has been proven that a correct diagnosis after profound data analysis could lead to a tremendous improvement in health costs^17^.

The numerous variables, the high number of unexplained cases reported, as shown in a cross-sectional study from NHANES 2007–2008^18^ and the misidentification of erythrocytosis seen in some cases^19^, make data collection complex. Misidentification of these cases may be consistent with the low reported data rate of SE cases at present.

Challenges of diagnosis between etiologies of erythrocytosis (PV versus SE) could be clarified after JAK2 somatic mutation genotyping is performed, as it prevails as a determinant tool in diagnosis^13^. In our cohort, the prevalence of the JAK2 V617F mutation was estimated to be 1.5% among cases of SE. Interestingly, we found that 3.7% of the SE patients had other clonal abnormalities of germline origin in the JAK2 gene. Even though the significance of these mutations and the risk of developing PV or leukemia phenotypes are unknown, tools to predict functional effects, such as the DANN score, could establish a probable risk of pathogenicity in some of these mutations^20,21^. With the development of more sensitive molecular diagnostic techniques, the prevalence of clonal hematopoiesis abnormalities seems to have increased in recent years and explains (at least partially) the differences seen across cohorts, including ours^22^.

While the number of patients without erythrocytosis was small in our study, the fact that bias can be introduced systematically in the identification of cases through disease code-based registries questions the validity of studies done with only disease codes as the strategy to identify cases.

This observation has been highlighted in previous publications, which increased the awareness of potential error sources and recommended code users to better evaluate the applicability and limitations of codes for their study of a particular disease or medical conditions^23^.

In our cohort, on the other hand, the code described as IE was given if the possibility of any etiology of erythrocytosis, whether primary or secondary, was excluded, and no further explanation was found for the presence of this phenomenon^24^. Consistent with the literature, aside from IE cases, the most commonly identified etiology of SE in this study was hypoxia, and approximately 24 (54.54%) patients had SE caused by OSA.

The main limitations in our study relate to its retrospective design of selected cases, importantly, the sample population analyzed for this study were referred to this institution which is cancer center, therefore our observations and findings are subjected to sampling bias. Moreover, our data analysis was restricted to patients who had JAK2 mutation molecular diagnostic studies. Furthermore, since several patients were evaluated as “second opinions” and their follow-up visits were carried out in various departments and medical centers, the etiology of erythrocytosis could not be established, accounting for approximately one-third of our cases. Another limitation includes the heterogeneity of the IE battery test used to evaluate secondary etiologies and the absence of follow-up data in several cases. Additional studies are needed for better understanding regarding the epidemiology of SE^25^.

The presence of germline variants described in this study are similar to observations from large European cohorts that have demonstrated the presence of clonal hematopoiesis in some general populations of patients with erythrocytosis without a diagnosis of PV^26^. In those cohorts, JAK-2 abnormalities were the most frequent among the identified clonal abnormalities.

The use of ICD data capture modalities is critical to accurately identify specific populations of interest to conduct retrospective research; however, limitations are associated with possible misdiagnosed cases. Even though in our study the majority of the patients were correctly classified, individual medical chart review may be necessary until a better classification method is reached to decrease the possibility of introducing bias in such studies. Furthermore, the relationship of the codes with the true clinical diagnosis, as well as the identification and systematic management of dual codes in software healthcare systems, to decrease case misidentification should be a priority to improve public health and clinical studies. Our findings suggest that it is important to evaluate cancer patients to offer appropriate care. As Sykes, et al. emphasizes, improvement on data registry and implementation of international registries are needed^8^. Guidelines and protocols for coding patients undergoing erythrocytosis evaluation can be of importance to prevent conflicting ICD diagnosis. Complementarily, health care providers should be trained to comply with ICD-10 practice, avoid coding for unconfirmed diagnoses and rather use a code for abnormal test results (ie. R.71)^27^ Larger-scale studies are needed to further identify the impact on health areas, as the outcomes of the above erythrocytosis research are hitherto unknown. Research based exclusively on ICD codes could have a potential impact on public health and patient care, and limitations must be weighed when research findings are conveyed.

## Data Availability

The datasets generated and/or analyzed during the current study are available from the corresponding author on reasonable request.
